# MassARRAY multigene screening combined with LDL-C and sdLDL-C detection for more favorable outcomes in type 2 diabetes mellitus therapy

**DOI:** 10.1186/s12920-021-00937-8

**Published:** 2021-03-17

**Authors:** Yong Tian, Junhong Wang, Yanxiao Liu, Xiangguang Luo, Ziying Yao, Xinjun Wang, Yuanyuan Zhang, Cheng Xu, Xiaoyu Zhao

**Affiliations:** 1 Department of Endocrinology and Metabolism, Pingdingshan People’s Hospital No.1, 117 Youyue Road, Pingdingshan, 467021 China; 2 Shanghai Biotecan Pharmaceuticals Co., Ltd, Shanghai Zhangjiang Institute of Medical Innovation, 180 Zhangheng Road, Shanghai, 200120 China; 3 State Key Laboratory of Genetic Engineerings, School of Life Sciences, Fudan University, 2005 Songhu Road, Shanghai, 200082 China; 4 Translational Medical Center for Stem Cell Therapy and Institute for Regenerative Medicine, Shanghai East Hospital, Shanghai Key Laboratory of Signaling and Disease Research, Frontier Science Center for Stem Cell Research, School of Life Sciences and Technology, Tongji University, 1239 Siping Road, Shanghai, 200092 China; 5 Wellbody Co., 160 Basheng Road, Shanghai, 200131 China

**Keywords:** MassARRAY, LDL-C, SdLDL-C, Type 2 diabetes mellitus, Cardiovascular disease

## Abstract

**Background:**

To determine the clinical value of multigene polymorphisms, LDL-C and sdLDL-C on T2DM therapy.

**Methods:**

In total, 352 T2DM patients before and after treatment and 48 healthy individuals were enrolled in this study. LDL-C and sdLDL-C were detected in 352 T2DM patients and 48 healthy individuals by Quantimetrix Lipoprint System. The 11 gene polymorphisms—*HTR3B* (rs2276307, A > G), *APOE* (rs7412, c.526C > T), *APOE* (rs429358, c.388 T > C), CYP2C9*3 (rs1057910, c.1075A > C), *KIF6* (rs20455, c.2155 T > C), *HMGCR* (rs17238540, T > G), *HMGCR* (rs17244841, A > T), *ABCB1* (rs2032582, A > C/T), *HTR7* (rs1935349, C > T), *SLCO1B1* (rs4149056, c.521 T > C), and *CETP* (rs708272, G > A)—were screened in these 352 T2DM patients by the Agena Bioscience MassARRAY system before therapy.

**Results:**

Genetic polymorphisms associated with T2DM and statin effects in pretreatment patients were detected, then results showed that all 11 genes had heterozygous mutation, and 7 genes had homozygous mutation in 352 T2DM patients, more specifically reflected that these gene polymorphisms were common in Chinese T2DM patients. LDL-C and sdLDL-C were detected before and after treatment, sdLDL mainly existed in T2DM patients, and T2DM patients had higher mean levels of sdLDL-C than healthy people. After pharmacotherapy, the coincidence rates of decreases in LDL-C and sdLDL-C levels were 88.35% (311/352) and 84.09% (296/352), consistent with patients in remission.

**Conclusions:**

Gene polymorphisms related to pharmacotherapy were common in Chinese T2DM patients. And the expression of LDL-C and sdLDL-C was consistent with the T2DM disease course. Combined multigene screening before therapy and LDL-C and sdLDL-C detection before and after therapy could better assist T2DM treatment.

**Supplementary Information:**

The online version contains supplementary material available at 10.1186/s12920-021-00937-8.

## Background

Diabetes mellitus is a metabolic disorder characterized by consistently elevated blood glucose [1, 2]. According to 2014 epidemiological data, approximately 8.3% of the world adult population has primary type 2 diabetes mellitus (T2DM) [3]. T2DM is associated with microvascular and macrovascular complications [4] that lead to cardiovascular or cerebrovascular issues [5]. Cerebral infarction in people with T2DM exhibits a different clinical pattern compared with that in patients without T2DM [6]. Therefore, T2DM is a powerful cardiovascular disease (CVD) risk factor [7].

Low-density lipoprotein cholesterol (LDL-C) is a major target for CVD prevention, and the UK Prospective Diabetes Study (UKPDS) demonstrated that LDL-C is a strong CVD risk factor in subjects with T2DM [7]. Many previous studies have shown that LDL phenotype may be divided into Pattern A and Pattern B according to particle size or density distributions, by a variety of laboratory methods measure, including analytical ultracentrifugation, density ultracentrifugation by vertical auto profile (VAP), segmented gradient gel electrophoresis (sGGE), tube gel electrophoresis (TGE), nuclear magnetic resonance (NMR), or ion mobility (IM) [8]. Pattern A consists of LDL-1 and LDL-2 subfractions, which named larger buoyant LDL (lbLDL). Pattern B consists of LDL-3 through LDL-7, which known as small dense LDL (sdLDL), and different LDL subfractions vary in their risk profiles [9–11]. Mean 10-year follow-up data in nondiabetic first-degree relatives (FDR) of consecutive patients with T2DM 30–70 years old showed that a higher LDL-C level was significantly associated with a higher risk of T2DM in high-risk individuals in Iran [12]. Total cholesterol, LDL-C, triglyceride and small dense LDL-C (sdLDL-C) levels were all significantly higher in diabetes patients than in nondiabetic individuals, and the elevation of serum sdLDL-C in patients with sustained hypertension suggests the establishment of atherogenic complications among diabetes patients [13, 14]. Therefore, the diagnosis and treatment of dyslipidemia is a cornerstone of diabetes mellitus management.

Statins are a common prescription medication for cholesterol reduction, and several intervention trials with statins have demonstrated the beneficial effect of lowering LDL-C in both primary and secondary CVD prevention, especially in subjects with T2DM [15, 16]. To ensure the T2DM treatment effect, genetic testing was recommended to patients before medication administration [17–24]. SNPs in the *HTR3B* and *HTR7* genes were significantly associated with the myalgia score and may affect the development of myalgia in statin-treated patients [17]. The *APOE* rs429358 and rs7412 polymorphisms were mainly associated with LDL-C and plasma total antioxidant capacity (T-AOC) levels (*p* < 0.05) [18]. Furthermore, CYP2C9*3 (1075A > C) was related to fluvastatin pharmacokinetics in Chinese populations [19]. Being a carrier of the c.2155 T > C variant of the *KIF6* gene negatively impacts patient responses to simvastatin, atorvastatin or rosuvastatin in terms of lipid-lowering effects [20]. In addition, *HMGCR* mutations cause a significant reduction in total cholesterol and LDL-C levels [21]. The *SLCO1B1* c.521 T > C variant significantly increased exposure to simvastatin acid by approximately 40% (*p* < 0.05) [22]. *ABCB1* (rs2032582: 2677G > T/A) was significantly associated with atorvastatin-induced liver injury (*p* = 0.00068) [23]. *CETP* rs708272 SNP together with statin therapy may show a favorable antiatherogenic effect [24].

**Fig. 1:**
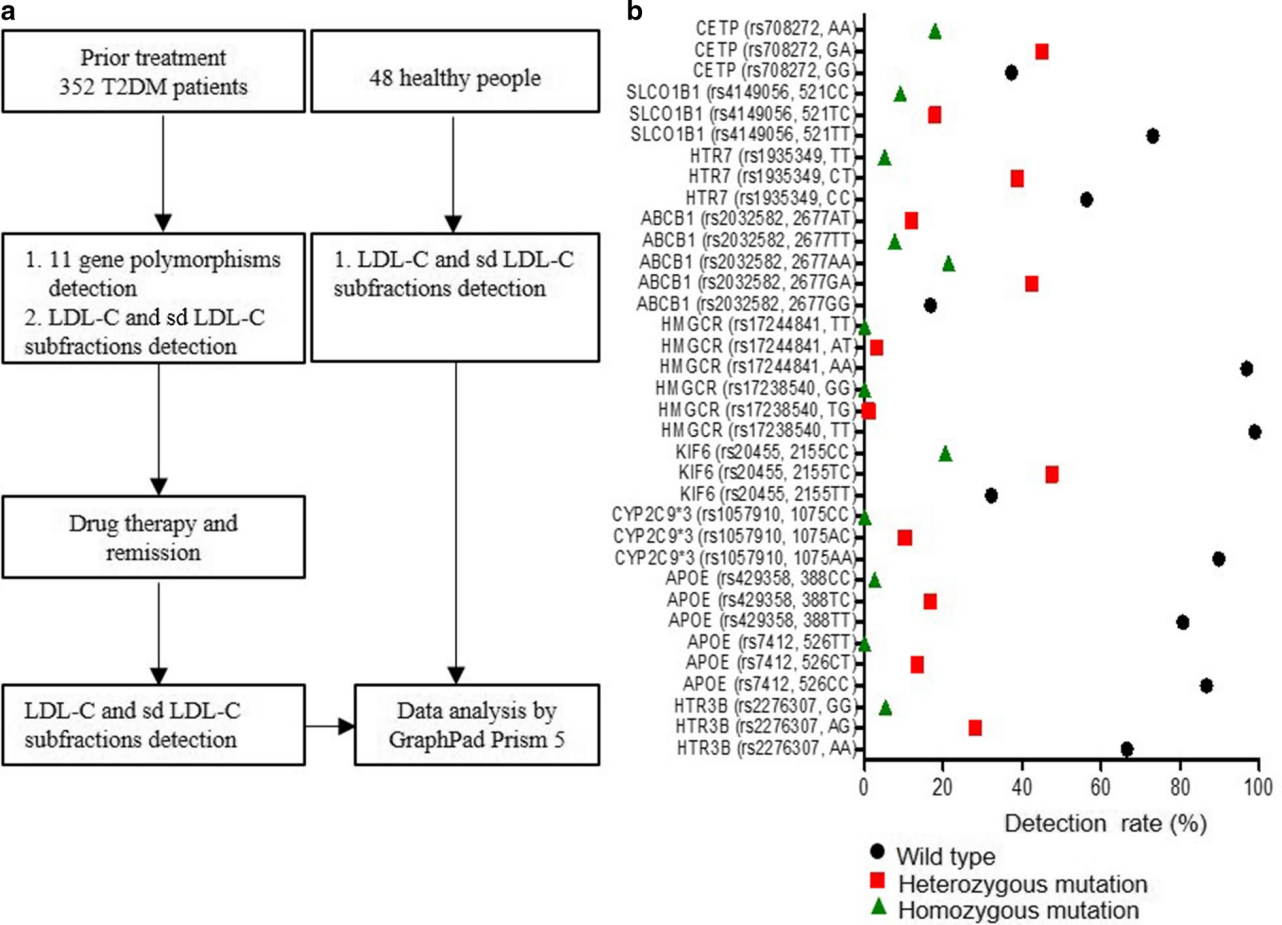
**a** Consort flow diagram. **b** The eleven gene polymorphisms detected in 352 T2DM patients (194 males and 158 females)

Although there are many reports about the excellent predictive performance of sdLDL-C for cardiovascular disease and T2DM, we still need to more precisely confirm the therapeutic effect of LDL-C or sdLDL-C in T2DM, and perhaps LDL subfractions have more precise clinical applications in T2DM. To improve T2DM therapy, multigene detection was performed before treatment by an Agena Bioscience MassARRAY system, which is an advanced detection system based on MALDI-TOF MS technology and can detect dozens of gene loci in one sample [25]. The study flowchart is shown in Fig. 1a. A total of 352 T2DM patients from Pingdingshan People’s Hospital No. 1 (Henan, China) were enrolled. Samples from T2DM patients underwent multigene detection before treatment, and LDL-C and sdLDL-C expression were evaluated before and after treatment with statins until the disease was improving. Finally, the data were analyzed by GraphPad Prism 5 statistical software.

## Methods

### Samples

A total of 400 subjects were recruited from Pingdingshan People’s Hospital No. 1 (Henan, China), including 352 T2DM patients (194 males and 158 females, 60.63 years mean age) and 48 healthy people (34 males and 14 females, 45.38 years mean age) (Table 1 and Additional file 1), from May 2018 to Jan 2020. All subjects signed an informed consent form before the study. Permission to use these samples was obtained from the Hospital Ethics Committees. Before detection, peripheral blood samples (1 ml each) were extracted from subjects and subjected to centrifugation at 800 × g for 10 min to obtain supernatant plasma samples (0.4 ml each) for LDL-C detection and peripheral blood cell sediment for MassARRAY SNP detection. The T2DM therapy guidance and remission evaluation criteria were following Guidelines for the Prevention and Treatment of Type 2 Diabetes in China (2017 Edition).

**Table 1:** Participant information (prior to treatment)

	T2DM patients (Mean ± SD)			Healthy people (Mean ± SD)		
	Male (n = 194)	Female (n = 158)	All (n = 352)	Male (n = 34)	Female (n = 14)	All (n = 48)
Age (year)	58.27 ± 12.44	63.52 ± 13.10	60.63 ± 12.99	45.82 ± 8.67	44.29 ± 6.07	45.38 ± 7.97
Body mass index (kg/m^2^)	26.31 ± 3.29	26.06 ± 4.79	26.20 ± 4.03	24.04 ± 1.94	22.96 ± 1.99	23.72 ± 1.99
LDL-1 (mg/dl)	17.68 ± 9.52	19.71 ± 9.82	18.59 ± 9.69	22.47 ± 11.59	25.93 ± 12.16	23.48 ± 11.74
LDL-2 (mg/dl)	21.33 ± 8.90	21.80 ± 8.90	21.54 ± 8.89	20.29 ± 5.71	21.64 ± 5.09	20.69 ± 5.52
LDL-3 (mg/dl)	12.79 ± 5.83	11.63 ± 6.50	12.27 ± 6.16	7.85 ± 4.55	7.86 ± 3.68	7.85 ± 4.28
LDL-4 (mg/dl)	6.65 ± 5.54	6.38 ± 5.35	6.53 ± 5.45	1.85 ± 2.72	1.71 ± 2.67	1.81 ± 2.68
LDL-5 (mg/dl)	3.47 ± 4.64	3.43 ± 4.14	3.45 ± 4.42	0	0	0
LDL-6 (mg/dl)	0.85 ± 1.41	0.71 ± 1.74	0.79 ± 1.55	0	0	0
LDL-7 (mg/dl)	0.29 ± 0.78	0.1 ± 0.45	0.20 ± 0.64	0	0	0
Total cholesterol (mmol/l)	4.45 ± 1.34	4.61 ± 1.04	4.54 ± 1.10	3.59 ± 0.72	3.96 ± 0.49	3.70 ± 0.68
Total triglycerides (mmol/l)	2.37 ± 1.80	2.86 ± 10.10	2.59 ± 6.89	1.42 ± 0.82	1.16 ± 0.42	1.34 ± 0.73
Plasma HDL-C (mmol/l)	1.12 ± 0.31	1.88 ± 8.34	1.46 ± 5.60	1.185 ± 0.27	1.33 ± 0.11	1.23 ± 0.24
Plasma LDL-C (mmol/l)	2.47 ± 0.86	2.58 ± 0.78	2.52 ± 0.83	2.04 ± 0.57	2.27 ± 0.44	2.11 ± 0.54
FBG (mmol/l)	8.42 ± 3.22	8.01 ± 3.25	8.24 ± 3.24	5.40 ± 1.08	4.88 ± 0.49	5.25 ± 0.97
SBP (mmHg)	141.95 ± 21.07	142.92 ± 18.68	142.39 ± 20.01	113.65 ± 12.66	109.64 ± 13.69	112.48 ± 12.95
DBP (mmHg)	86.19 ± 12.38	80.17 ± 12.04	83.49 ± 12.57	76.18 ± 7.24	75.86 ± 6.72	76.08 ± 7.03
Heart rate (bpm)	83.60 ± 14.22	82.55 ± 13.10	83.13 ± 13.72	80.29 ± 7.69	82.50 ± 7.06	80.94 ± 7.50
SCr (μmol/l)	74.50 ± 33.83	58.22 ± 21.33	67.17 ± 29.96	74.97 ± 11.15	60.93 ± 5.86	70.88 ± 11.76
Hcy (μmol/l)	14.20 ± 8.92	10.77 ± 3.82	12.66 ± 7.29	13.50 ± 3.49	11.76 ± 2.96	13.00 ± 3.41
GHb (%)	8.41 ± 1.77	8.59 ± 2.18	8.49 ± 1.96	4.94 ± 0.53	4.97 ± 0.55	4.94 ± 0.53

### LDL-C detection

First, total cholesterol, total triglycerides, plasma HDL-C, and plasma LDL-C were tested by PTS PANELS Lipid Panel Test Strips (PTS diagnostics, NO: PTS-1710) and analyzed by CardioChek® PA (PTS diagnostics, USA) for LDL-C subfraction auxiliary analysis. Then, LDL-C subfraction detection was processed by a Quantimetrix Lipoprint System LDL Subfraction Kit (REF48-7002, Manhattan Beach, CA, USA) [26]. In detail, the gel tubes were first removed from the jar and placed in the preparation rack, and the storage buffer was completely removed from the top of the gels. Then, 25 μl plasma samples were added to each tube, and 200 μl of Lipoprint loading gel was put into each tube. Then, a strip of Parafilm was placed between the gel tubes and preparation rack cover. The loading gel with the specimen was mixed by reverse blending the preparation rack several times.

This loading gel was photopolymerized for 30 min by the preparation light, and then each gel tube was removed from the preparation rack and carefully inserted into the silicone adapter of the upper chamber. One hundred milliliters of electrolyte buffer solution were placed in the lower chamber, while 200 ml of this solution was placed in the upper chamber. The electrophoresis chamber lid was put in place and connected to the power source. The power source was adjusted to deliver a current of 3 mA per gel tube, and the samples were electrophoresed at 500 V for 60 min. The power was turned off after the electrophoresis was complete, the chamber lid was removed, and the electrolyte buffer in the upper chamber was discarded. Finally, the gel tube was put into the preparation rack and analyzed.

### MassARRAY SNP detection

The MassARRAY iPLEX Gold multiple genotyping analysis system (Agena Bioscience, Inc.) was used, and the reagents contained Agena PCR reagent, Agena SAP reagent, and Agena iPLEX reagent. For the test details, peripheral blood cell sediment was extracted following the reagent’s protocol (TIANGEN, DP348). PCR mixtures were obtained via the Agena PCR reagent set and start PCR procedures, and then these mixtures were treated with shrimp alkaline phosphatase (SAP). After extending the reaction, the samples underwent desalination processing and dispensing on the chip, were then analyzed by MALDI-TOF MS. MassARRAY primers are shown in Table 2.

**Table 2:** MassARRAY primers

Gene	SNP_ID	Forward primer sequence	Reverse primer sequence	UEP sequence
*HTR3B*	rs2276307	ACGTTGGATGAAGTCCCTGTTCTTGGGTGA	ACGTTGGATGCTTTGGCCTTCTCTCTTGGG	CTCTTGGGCCAAGGA
*APOE*	rs7412	ACGTTGGATGGCCCCGGCCTGGTACACTG	ACGTTGGATGACCTGCGCAAGCTGCGTAA	CGATGACCTGCAGAAG
*APOE*	rs429358	ACGTTGGATGGAGCATGGCCTGCACCTCG	ACGTTGGATGCTGTCCAAGGAGCTGCAGG	ATGACATGGAGGACGTG
CYP2C9*3	rs1057910	ACGTTGGATGATGCAAGACAGGAGCCACAT	ACGTTGGATGTGTCACAGGTCACTGCATGG	GTGGGGAGAAGGTCAA
*KIF6*	rs20455	ACGTTGGATGCCGGTGAGTTCTCACCTTAC	ACGTTGGATGCGATCACACGAAGCCATTTC	CTGACTCCCAGCATGAA
*HMGCR*	rs17238540	ACGTTGGATGGGACACAATGGATTAGGCTG	ACGTTGGATGGAGACTATGTATCACTCACC	GGTCTTTTCCAAACTCTTT
*HMGCR*	rs17244841	ACGTTGGATGCACACCATTGCACATTGCAC	ACGTTGGATGCAGGTATTCAAGATACAAAG	AAGTATGATTGTAATATAAAGGATTT
*ABCB1*	rs2032582	ACGTTGGATGGTCTGGACAAGCACTGAAAG	ACGTTGGATGAGTAAGCAGTAGGGAGTAAC	CCTCTGACTCACCTTCCCAG
*HTR7*	rs1935349	ACGTTGGATGGTGTCTGTGGTCAGGTGATA	ACGTTGGATGTATTTCCTTGGCTGCCAGTC	AAATAGATTGTCCAGACATGA
*SLCO1B1*	rs4149056	ACGTTGGATGAATCTGGGTCATACATGTGG	ACGTTGGATGCCAATGGTACTATGGGAGTC	CCCAAGCATATTACCCATGAAC
*CETP*	rs708272	ACGTTGGATGTGTCTGAGACCCAGAATCAC	ACGTTGGATGTCTTTACCCCCTGACTCAAC	CGGCGCAGATCTGAACCCTAACT

### Statistical analysis

**Fig. 2:**
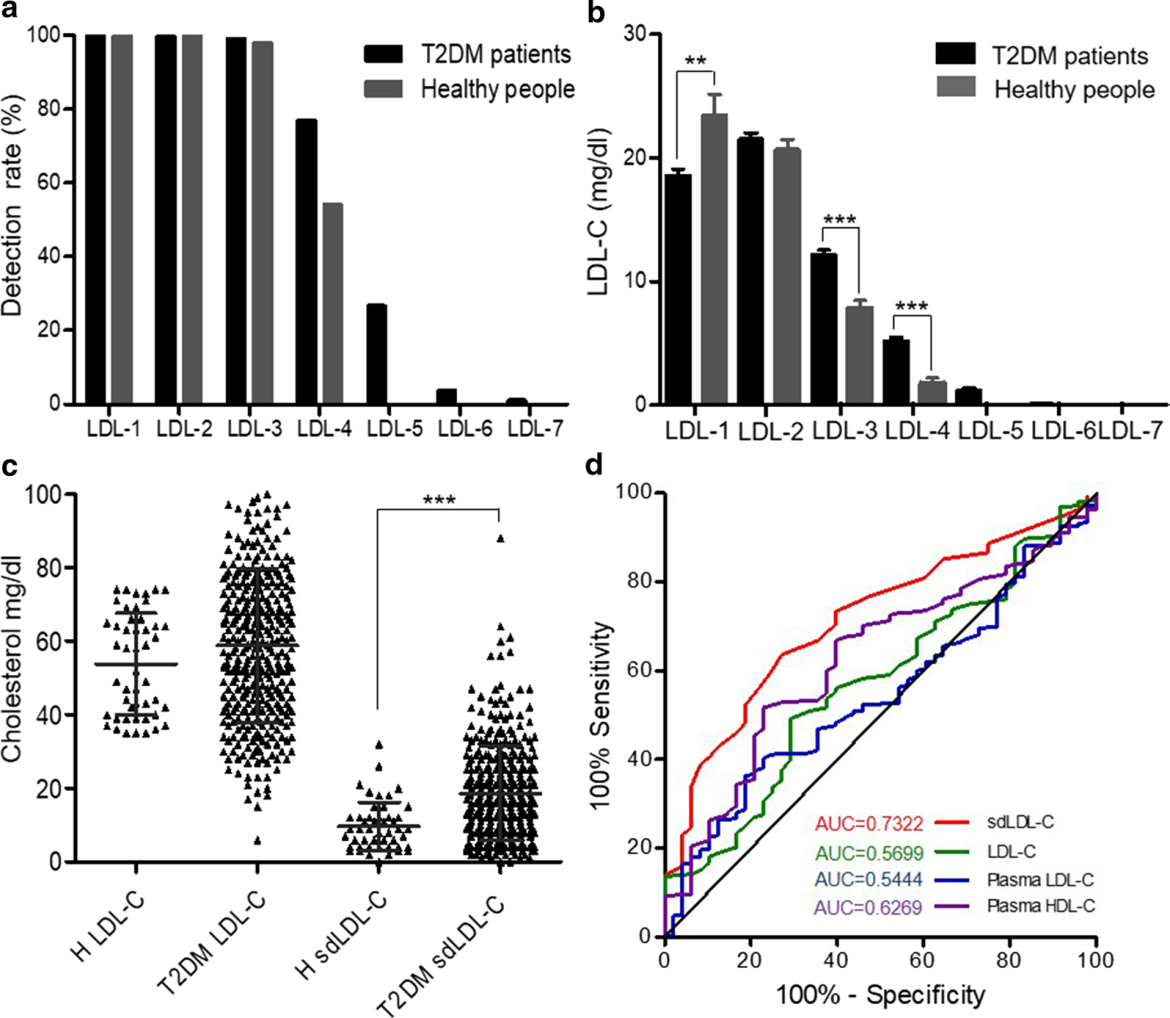
LDL-C subfraction detection. **a** Detection rate of LDL-1 to LDL-7 subfractions in T2DM patients (n = 352) and healthy people (n = 48). **b** Mean amount of LDL-1 to LDL-7 subfractions in T2DM patients (n = 352) and healthy people (n = 48). **c** Expression of LDL-C and sdLDL-C in 352 T2DM patients before treatment and 48 healthy people. **d** ROC analysis of 352 T2DM patients and 48 healthy people for sdLDL-C, LDL-C, plasma LDL-C and plasma HDL-C. ANOVA, ***p < 0.001, **p < 0.01

**Fig. 3:**
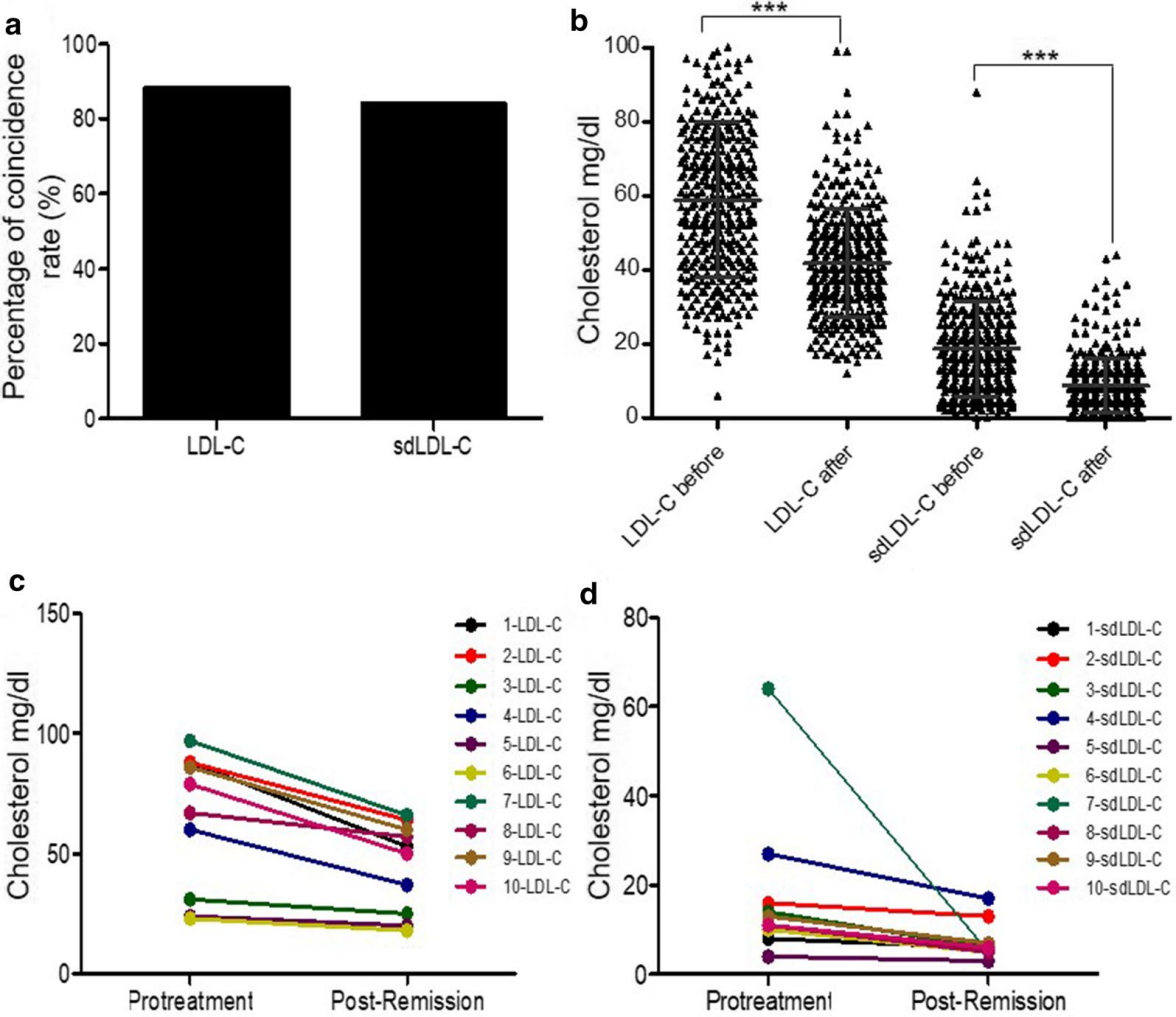
LDL-C and sdLDL-C monitoring in T2DM patients. **a** A total of 352 T2DM patients (194 males and 158 females) underwent detection. **b** Expression of LDL-C and sdLDL-C in 352 T2DM patients before treatment and after treatment. ANOVA, ***p < 0.001. **c** LDL-C was detected in 10 T2DM patients (6 males and 4 females) before treatment and after treatment. **d** sdLDL-C was detected in 10 T2DM patients (6 males and 4 females) before treatment and after treatment. The numbers 1 to 10 represent patients 1 to 10

Data were analyzed by GraphPad Prism 5 statistical software (GraphPad Software, Inc., San Diego, CA, USA). Figure 1b of 11 gene polymorphisms analysis of 352 T2DM patients was by GraphPad Prism 5 statistical software. Figure 2a–c LDL-C subfraction detection were analysed by GraphPad Prism 5 statistical software and *p* value of < 0.05 was considered to indicate a statistically significant difference. Figure 2d of ROC analysis for 352 T2DM patients and 48 healthy people was by GraphPad Prism 5 statistical software. Figure 3 LDL-C and sdLDL-C monitoring in T2DM patients was analysed by GraphPad Prism 5 statistical software, and *p* value of < 0.05 was considered to indicate a statistically significant difference.

#### Results

### Multitudinous gene polymorphisms in Chinese T2DM patients

Many studies have shown that gene polymorphisms influence T2DM therapeutic effects [17–24]. SNPs in the *HTR3B*, *HTR7* or *ABCB1* genes were associated with myalgia or liver injury [17, 23]. *APOE* and *HMGCR* mutations were associated with LDL-C levels [18, 21]. *CETP*, *KIF6*, *SLCO1B1*, and CYP2C9*3 were related to the statin effect [19, 20, 22, 24]. To check genetic polymorphisms in Chinese T2DM patients, *HTR3B* (rs2276307, A > G), *APOE* (rs7412, c.526C > T), *APOE* (rs429358, c.388 T > C), CYP2C9*3 (rs1057910, c.1075A > C), *KIF6* (rs20455, c.2155 T > C), *HMGCR* (rs17238540, T > G), *HMGCR* (rs17244841, A > T), *ABCB1* (rs2032582, c.2677G > T/A), *HTR7* (rs1935349, C > T), *SLCO1B1* (rs4149056, c.521 T > C), and *CETP* (rs708272, G > A) were detected by the MassARRAY system before patients underwent statin therapy (Fig. 1b and Table 2).

All 11 mutation loci were checked; and we found all 11 genes had heterozygous mutation, and 7 genes had homozygous mutation in T2DM patients (Fig. 1b and Additional file 2). *KIF6* (rs20455, c.2155 T > C) had the highest heterozygous mutation (47.44%, n = 167), while ABCB1 (rs2032582, c.2677G > T/A) had the highest homozygous mutation (21.31%, n = 75) in these patients (Fig. 1b). These results reflected that gene polymorphisms were common in Chinese T2DM patients, and that gene polymorphism detection before treatment had a certain significance for patients. For instance, *SLCO1B1* was related to myopathy; this test result showed that *SLCO1B1* (rs4149056, 521CC) was harbored by 9.09% (n = 32) of patients, and patients with this genotype had a high risk of myopathy and rhabdomyolysis [27]. *SLCO1B1* (rs4149056, 521TC) was carried by 17.90% (n = 63) of patients, and these patients had a medium risk of myopathy and rhabdomyolysis, with statins tolerated at a medium dose (Fig. 1b). 2019 ESC/EAS guidelines for the management of dyslipidemia indicates that *CYP2C8*, *CYP2C9*, *CYP2C19*, and *CYP2D6* are frequently involved in the metabolism of statins [28]. In this study, CYP2C9*3 (rs1057910, c.1075A > C) AA, AC, and CC genotypes were carried by 89.77%, 10.23%, and 0% of the patients, respectively, and the AC genotype was associated with a high risk of myopathy after fluvastatin was used [19]. Therefore, genotype evaluation is strongly necessary evaluation for T2DM patients before treatment therapy.

### sdLDL-C subfractions had superior property in T2DM screening.

Previous studies found that sdLDL-C levels were significantly higher in diabetes patients than in nondiabetic individuals [13, 14]. To determine the expression of LDL-C and sdLDL-C in T2DM patients and healthy people, the Quantimetrix Lipoprint system was used for plasma sample analysis following the protocol. In total, 400 subjects were analyzed, including 352 T2DM patients and 48 healthy people. The detection rates of the LDL-1, LDL-2, LDL-3, LDL-4, LDL-5, LDL-6, and LDL-7 subfractions in T2DM patients were 100%, 99.72%, 99.15%, 76.99%, 26.70%, 3.69%, and 1.14%, while those in healthy people were 100%, 100%, 97.92%, 54.17%, 0%, 0%, and 0%, respectively (Fig. 2a). The strong CVD risk factor LDL-5 to LDL-7 existed in T2DM patients, were not found in healthy people (Fig. 2a).

Then LDL-C expression were analyzed, the mean amounts of LDL1-C to LDL7-C in T2DM patients were 18.59 mg/dl, 21.54 mg/dl, 12.27 mg/dl, 6.53 mg/dl, 3.45 mg/dl, 0.79 mg/dl, and 0.20 mg/dl, while these subfractions in healthy people were 23.48 mg/dl, 20.69 mg/dl, 7.85 mg/dl, 1.81 mg/dl, 0 mg/dl, 0 mg/dl, and 0 mg/dl (Fig. 2a and Table 1). Further analysis revealed that Pattern A, which consisted by LDL-1 and LDL-2, had no obvious difference between T2DM patients and healthy people. Predictable, Pattern B, composited by LDL3-C to LDL7-C, which was known as sdLDL had higher expression in T2DM patients than healthy people and had obvious differences (*p* < 0.001) (Fig. 2c). This result further confirmed that sdLDL was the high-risk T2DM factor.

To determine screening effect for T2DM, sdLDL-C, LDL-C, plasma LDL-C and plasma HDL-C of the 352 T2DM patients and 48 healthy people were analyzed by receiver operating characteristic (ROC) curve analysis. The area under the curve (AUC) of these four biomarkers in 352 T2DM patients and 48 healthy people were 0.7322, 0.5699, 0.5444 and 0.6269 respectively (Fig. 2d), and sdLDL-C had the highest value compared to the other three biomarkers. Therefore, sdLDL-C was highly expressed in T2DM patients and had superduper screening effect.

### LDL-C and sdLDL-C had excellent monitoring performance on T2DM therapy

To verify the clinical value of LDL-C and sdLDL-C on T2DM therapy monitoring, these two biomarkers were detected and analyzed for 352 T2DM patients (194 males and 158 females) on the condition of prior treatment and after treatment remission. Total 352 T2DM patients were suffered drug therapy, and the guidance and remission evaluation criteria were referencing Guidelines for the Prevention and Treatment of Type 2 Diabetes in China (2017 Edition). Before treatment and anesis after treatment LDL-C and sdLDL-C were analyzed, after 352 T2DM patients alleviating, coincidence rate of decreasing LDL-C accounted for 88.35% (311/352), while coincidence rate of decreasing sdLDL-C was 84.09% (296/352), and there was no significant difference between these two values (Fig. 3a).

Next, the expression levels of total 352 T2DM patients before and after treatment of LDL-C and sdLDL-C were analyzed, found the expression of posttreatment LDL-C and sdLDL-C were reduced compared with prior treatment, and had significant difference (*p* < 0.001) (Fig. 3b). The results showed that LDL-C and sdLDL-C were good indicators for T2DM treatment effect evaluation. In order to accurately reflect the expression changes of LDL-C and sdLDL-C in the process of disease remission, 10 T2DM patients (6 males and 4 females) were randomly selected. Both the expression levels of LDL-C and sdLDL-C were decreased after disease remission (Fig. 3c, d). Therefore, LDL-C and sdLDL-C may be used as excellent monitoring biomarkers for T2DM therapy.

## Discussion

Statins are currently effective in the treatment of T2DM and lowering blood lipids [15, 16, 29]. Polymorphisms of multiple genes that may be associated with therapeutic efficacy were detected by the Agena Bioscience MassARRAY system before patient treatment [25]. The research showed that CYP2C9*3 (1075A > C) is concerned with the fluvastatin pharmacokinetics in Chinese individuals [19], *HMGCR* mutations cause total cholesterol and LDL-C levels to decrease [21], and the *SLCO1B1* c.521 T > C variant distinctly increases exposure to simvastatin acid [22].

In this study, 11 gene mutation loci were checked, we found all 11 genes had heterozygous mutation, 7 genes had homozygous mutation (Fig. 1b). *KIF6* (rs20455, c.2155 T > C) had the highest heterozygous mutation (47.44%), *ABCB1* (rs2032582, c.2677G > T/A) had the highest homozygous mutation (21.31%) in these 352 T2DM patients (Fig. 1b). In addition, 17.90% (n = 63) *SLCO1B1* (rs4149056, 521TC), 9.09% (n = 32) *SLCO1B1* (rs4149056, 521CC) existed in these patients (Fig. 1b), and patients with these genotypes had a medium and high risk of myopathy and rhabdomyolysis, respectively [27]. 10.23% patients had CYP2C9*3 (rs1057910, 1075AC) and had a high risk of myopathy after fluvastatin was used [19] (Fig. 1b). The numerous mutations identified suggest that polymorphism testing is necessary for T2DM patients before treatment to achieve the best therapeutic schedule.

Previous researches showed that diabetes patients had higher sdLDL-C level [12, 13], in our study, this result was confirmed. The LDL-C and sdLDL-C subfractions of 352 T2DM patients and 48 healthy people were analyzed, Pattern A had no obvious difference between T2DM patients and healthy people while Pattern B had higher expression in T2DM patients and had obvious differences (*p* < 0.001) (Fig. 2c). This result was consistented with previous research [9–11]. Based on ROC analysis, sdLDL-C had the best screening performance in distinguishing T2DM patients from healthy people (AUC = 0.7322), compared to LDL-C, plasma LDL-C and plasma HDL-C (Fig. 2d). This consequence further demonstrated that sdLDL-C was is an effective indicator for T2DM risk monitoring. Meanwhile, there were 48 healthy samples and fewer than T2DM samples. Even so, the LDL-C and sdLDL-C detection results of these 48 healthy people were similar to our previous study, which included 100 healthy samples (the result didn't show). Hence, despite healthy samples were fewer, it didn't affect the accuracy of the result.

Then the therapy monitoring efficacy of LDL-C and sdLDL-C were explored, for patients whose treatment was effective had lower LDL-C levels [30]. After treatment following Guidelines for the Prevention and Treatment of Type 2 Diabetes in China (2017 Edition) and patients were in remission, the coincidence rates of decreases in LDL-C and sdLDL-C were 88.35% (311/352) and 84.09% (296/352), respectively, in contrast to the expression changes in LDL-C and sdLDL-C (Fig. 3a), consistent with previous studies [31–33]. We also performed dynamic analysis of LDL-C and sdLDL-C expression in 10 randomly selected patients; although reduction differed, all patients showed a downward trend (Fig. 3c, d). Therefore, LDL-C and sdLDL-C could be effective monitoring indicators for T2DM.

### Conclusion

In conclusion, combined multigene screening and LDL-C and sdLDL-C subfractions detection could help effectively adjust the therapeutic strategy for T2DM patients before treatment and help monitor the therapeutic effect after treatment.

## Supplementary Information

**Additional file 1**. Cohort baseline and LDL monitoring results. I) The baseline information of our cohort. II) The LDL baseline of patients before drug treatment. III) The LDL level of patients after drug treatment

**Additional file 2**. Gene polymorphisms result of our cohort. Gene polymorphism data of patients at baseline timing

## Data Availability

The datasets generated and/or analysed during the current study are available in the Supplementary information, including Additional file 1 (Cohort baseline and LDL monitoring results) and Additional file 2 (Gene polymorphisms result of our cohort). All authors declare that data and any supporting material regarding this manuscript can be requested at any time.

## Abbreviations

T2DM: Type 2 diabetes mellitus
LDL-C: Low-density lipoprotein cholesterol
sd LDL-C: Small dense LDL-C
CVD: Cardiovascular disease
AUC: Area under the curve
ROC: Receiver operating characteristic
